# Digital Health and Learning in Speech-Language Pathology, Phoniatrics, and Otolaryngology: Survey Study for Designing a Digital Learning Toolbox App

**DOI:** 10.2196/34042

**Published:** 2022-04-27

**Authors:** Yuchen Lin, Martin Lemos, Christiane Neuschaefer-Rube

**Affiliations:** 1 Clinic for Phoniatrics, Pedaudiology & Communication Disorders University Hospital and Medical Faculty, Rheinisch-Westfaelische Technische Hochschule Aachen Aachen Germany; 2 Audiovisual Media Center (AVMZ) University Hospital and Medical Faculty, Rheinisch-Westfaelische Technische Hochschule Aachen Aachen Germany

**Keywords:** digital learning, mLearning, mHealth, speech-language pathology, phoniatrics, otolaryngology, communication disorders, mobile phone

## Abstract

**Background:**

The digital age has introduced opportunities and challenges for clinical education and practice caused by infinite incoming information and novel technologies for health. In the interdisciplinary field of communication sciences and disorders (CSD), engagement with digital topics has emerged slower than in other health fields, and effective strategies for accessing, managing, and focusing on digital resources are greatly needed.

**Objective:**

We aimed to conceptualize and investigate preferences of stakeholders regarding a *digital learning toolbox*, an app containing a library of current resources for CSD. This cross-sectional survey study conducted in German-speaking countries investigated professional and student perceptions and preferences regarding such an app’s features, functions, content, and associated concerns.

**Methods:**

An open web-based survey was disseminated to professionals and students in the field of CSD, including speech-language pathologists (SLPs; German: Logopäd*innen), speech-language pathology students, phoniatricians, otolaryngologists, and medical students. Insights into preferences and perceptions across professions, generations, and years of experience regarding a proposed app were investigated.

**Results:**

Of the 164 participants, an overwhelming majority (n=162, 98.8%) indicated readiness to use such an app, and most participants (n=159, 96.9%) perceived the proposed app to be helpful. Participants positively rated app functions that would increase utility (eg, tutorial, quality rating function, filters based on content or topic, and digital format); however, they had varied opinions regarding an app community feature. Regarding app settings, most participants rated the option to share digital resources through social media links (144/164, 87.8%), receive and manage push notifications (130/164, 79.3%), and report technical issues (160/164, 97.6%) positively. However, significant variance was noted across professions (*H*_3_=8.006; *P=*.046) and generations (*H*_3_=9.309; *P=*.03) regarding a username-password function, with SLPs indicating greater perceived usefulness in comparison to speech-language pathology students (*P*=.045), as was demonstrated by Generation X versus Generation Z (*P*=.04). Participants perceived a range of clinical topics to be important; however, significant variance was observed across professions, between physicians and SLPs regarding the topic of diagnostics (*H*_3_=9.098; *P*=.03) and therapy (*H*_3_=21.236; *P*<.001). Concerns included technical challenges, data protection, quality of the included resources, and sustainability of the proposed app.

**Conclusions:**

This investigation demonstrated that professionals and students show initial readiness to engage in the co-design and use of an interdisciplinary digital learning toolbox app. Specifically, this app could support effective access, sharing, evaluation, and knowledge management in a digital age of rapid change. Formalized digital skills education in the field of CSD is just a part of the solution. It will be crucial to explore flexible, adaptive strategies collaboratively for managing digital resources and tools to optimize targeted selection and use of relevant, high-quality evidence in a world of bewildering data.

## Introduction

### Background

Mobile devices are rapidly revolutionizing the means of communication, learning, and health care. Since the emergence of new technologies and devices such as smartphones, tablets, PDAs, smartwatches, and laptop computers among others in the past decades, the adoption of such technologies in the health sciences and medical education has been increasingly explored [1-3]. Specifically, the use of mobile devices for patient care, both by patients (eg, health apps) and by clinical professionals (eg, patient monitoring tools, telemedicine, and teletherapy) and for research and education purposes (eg, reference apps) has increased [4-6]. This has especially been prevalent considering the COVID-19 pandemic, which has pushed investigation into creating and improving such solutions to the forefront [7-9]. In this context, the term *mobile health* or *mHealth* has emerged to describe the broad spectrum of information-communication technology for medical and public health practices supported by mobile devices, whereas the terms *mobile learning* or *mLearning* have evolved to describe the use of mobile devices to deliver educational content for preclinical, clinical, or specialty training and continuing education or professional development [10,11]. Given that most clinical professionals and students own such mobile technology and the evidence that health care students prefer web-based resources as their primary source of clinical information, the use of mobile medical apps as reference tools is becoming the norm as opposed to an exception [12-14]. As implementation of such technologies and apps continues to increase, it is evident that the future of medicine will inevitably require health professionals to flexibly include media literacy, digital knowledge, and skills into part of their professional scopes of practice.

In the field of communication sciences and disorders (CSD), investigation into digital solutions such as mobile health and mobile learning apps is also becoming popular, although at a slower pace than in other medical fields [15,16]. As an interdisciplinary field concerned with treating the estimated 1 billion people worldwide living with a disability often affecting their speech, language, hearing, voice, or ability to functionally communicate, the field strongly relies on the effective, coordinated efforts of speech-language pathologists (SLPs), phoniatricians, and otolaryngologists among others [17]. In the ever-evolving health care environment, digital solutions especially have the potential to optimize interdisciplinary care and collaboration, which has been identified as a key component to *futureproofing* health care, in other words, designing adaptable solutions for even when technology progresses [18-21]. Thus, exploration of digital resources across disciplines can be useful. In addition to improvements to well-established digital technologies in the field such as augmentative and alternative communication devices, hearing aids, and cochlear implants, mobile technologies are beginning to revolutionize alternative methods of service delivery (eg, telerehabilitation and telepractice) and treatment material (eg, digital therapy or medical apps) and are increasingly empowering patients to engage in their own health management to a greater extent [22-25]. Furthermore, there is some evidence that mobile app technology through smartphones and tablets can improve performance in both speech-language pathology graduate students and otolaryngology and phoniatrics residents when explicitly trained or used as knowledge building (eg, case scenarios, simulations, and question banks) or resource sharing tools [24,26-28]. Although the uncountable and increasing number of apps is impressive, it may be beginning to pose a challenge to meaningful, evidence-based, clinical decision-making and learning [24,29]. In response, digital tools are emerging to provide clinical professionals with faster and easier access to preassessed, evidence-based, psychometrically sound assessments for clinical purposes. For example, the NIH Toolbox for the Assessment of Neurological and Behavioral Function from the National Institutes of Health specifically serves as a comprehensive and portable digitized battery of measures for clinicians to assess and track motor, emotional, sensory, and cognitive functions. By using the benefits of big data, the system can transmit 15,000 data points in ≤7 minutes and has been investigated in >600 studies [30]. Similarly, the PROMIS (Patient-Reported Outcomes Measurement Information System) iPad app, which has been investigated in >2000 studies so far, allows for the monitoring of physical, mental, and social health in adults [31]. Similarly, Torous and Vaidyam [32] designed the mindLAMP (learn, assess, manage, and prevent) mental health app with the intent of more comprehensively addressing multiple user needs; importantly, they emphasized that collaboratively designed comprehensive platforms in the form of a *digital health technology toolbox* can help to eliminate the need for single-purpose apps and could potentially maximize utility and user uptake. However, importantly, such comprehensive, data-backed tools specifically within CSD are scarce.

Moreover, even though such tools are available, the influx of digital resources appears to be undermined because many professionals and students are reportedly unfamiliar with such tools and are not confident in their knowledge and skills pertaining to digital health and clinical resources [33-35]. Although >80% of health professionals surveyed in a European Health Parliament questionnaire reported feeling unprepared for technological developments in health care, 60% of students surveyed across 39 countries similarly felt inadequately trained for the digitalizing health care environment, citing lack of digital skills training and knowledge of digital tools and resources as causes [34,35]. Although professionals and students in CSD have demonstrated interest in increased digital topics in clinical training and continuing education, studies have demonstrated that only approximately 36% to 41% of speech-language pathology academic training programs in the United States explicitly incorporated telepractice apps as part of their curricula, and digital skills have not consistently been an integral component of medical otolaryngology or phoniatric specialty training programs [28,36,37]. Moreover, the increasing number of digital clinical resources and tools is accompanied by a concern of information and data overload, which can make judging the relevance and usefulness of information more difficult. It has been suggested that rather than the issue being an influx in digital information and data, it may be that traditional strategies for managing and evaluating information have not progressed at the same pace as the production of information [38,39]. Furthermore, this can make it difficult to assess the quality of digital clinical resources, many of which have not been peer reviewed or have unknown publishers [40,41].

### Objectives

Thus, as digital resources continue to grow exponentially [10,11], it is becoming increasingly clear that professionals and students require digital skills and media literacy training and strategies for sorting through and critically evaluating the quality of digital resources that already exist. Therefore, having an up-to-date library of field-relevant, interdisciplinary, digital learning and therapy tools, which could be collaboratively expanded upon and accessed across multiple platforms or devices in the form of an app, could be useful. Expanding upon the concept of a multiple-use *digital health technology toolbox* previously mentioned by Torous and Vaidyam [32], we proposed that a digital resource library app focused on resource sharing rather than clinical assessment—what we have termed a *digital learning toolbox* (DLT)—could be useful. Specifically, such a tool could help to spark discussion regarding quality assessment, usefulness, and areas of need for existing digital resources. To support the future development of such a digital resource library app with maximized user-centered design, our study aimed to gain insights into the perceptions and preferences of valuable stakeholders, specifically professionals and students in speech-language pathology, phoniatrics, and otolaryngology in German-speaking countries (mainly Germany, Austria, and Switzerland). Specifically, we aimed to determine the interest in such an app and identify potential concerns and desired features, functions, or content through a structured questionnaire, which was disseminated to the speech-language pathology, phoniatrics, and otolaryngology professional and academic communities. Differences across professions, generations, and years of experience were also explored.

## Methods

### Overview

This survey study was conducted in accordance with the CHERRIES (Checklist for Reporting Results of Internet E-Surveys) guidelines [42]. This survey was the second part of a large survey study. In the first part, knowledge, use, attitudes, and preferences toward digital health and learning of current students and professionals across the interdisciplinary fields of speech-language pathology, phoniatrics, and otolaryngology in the German-speaking countries were investigated. The results of the first section have already been published in a separate article to maximize the depth of analysis [43]. This study focuses separately on professional and student attitudes and preferences regarding a proposed DLT app. The target populations of the proposed app were professionals and students in CSD, including physicians (phoniatricians and otolaryngologists), SLPs, medical students, and speech-language pathology students. The app would serve as an interdisciplinary, collaborative library of open-source digital learning and therapy tools. It would include content relating to anatomy and physiology, pathology, diagnostics, therapy, professional practice issues, and networking. Moreover, this proposed app could include functions such as introductory tutorial; filter functions based on content, language, or source among others; tool rating function; glossary; and app community. Additional settings for increased usability such as a tool sharing function, tool organization function, notification management, and technical error reporting could also be incorporated. To co-design the app to be maximally useful, professionals and students were asked to rate and provide their input on desired content, functions, and settings. Survey screens and a narrative explanation are included in Multimedia Appendix 1.

### Ethics Approval

The Ethics Committee of the Medical Faculty at the University Hospital of the Rheinisch-Westfälische Technische Hochschule Aachen University (EK 188/20) determined that the study did not require a full data protection impact assessment as the questionnaire used in the study was fully anonymous. Demographic information regarding profession, years of experience, generation, and sex was collected. Participation was voluntary and could be ended at any time.

### Participants and Recruitment

An invitational letter and flyer containing a link to the open survey was shared with professional regulating bodies and university clinical programs in speech-language pathology, phoniatrics, and otolaryngology and with relevant open student and professional groups on Facebook within German-speaking countries. To participate in the survey, participants had to be one of the following: (1) physician in phoniatrics or otolaryngology, (2) SLP, (3) medical student, or (4) speech-language pathology student. Before beginning the survey, participants were prompted to read through detailed study background, aims, procedures, anonymous data to be collected, data protection policies, and contact persons and were required to provide informed consent before proceeding. Other than demographic information including profession, years of experience, generation, and sex, no personal information was collected, and no incentives for participation were offered.

### Platform

The web-based survey was hosted on university-licensed LimeSurvey (version 4.3.14+200826; LimeSurvey GmbH), a web-based statistical survey web application that conforms to the required data security legislation dictated by the German Federal Data Protection Act, the European Data Protection Directive 95/46/EC, and the European General Data Protection Regulation [44]. To prevent repeated access to the survey, unique survey visitors were tracked by cookies as allowed per the participant’s browser settings, but no IP addresses were saved. Cookies were set at the start of the survey and were valid for the LimeSurvey default of 365 days.

### Survey Design and Content

An interdisciplinary team (the authors) consisting of an SLP (YL), phoniatrician and otolaryngologist (CNR), and instructional designer (ML) developed, pretested, and cross-checked a semistructured anonymous questionnaire to ensure comprehensibility and appropriateness of the survey questions. This second part of the survey contained 15 questions pertaining to sociodemographic information and attitudes and preferences regarding a proposed digital resource library app, which we have termed a DLT. There were 12 screens with 1 to 4 questions displayed per page, including the initial page with participant information on which the participant had to give consent before proceeding. The survey contained the following question types: yes or no questions, multiple-answer questions (with a free-text response option), arrays with Likert scale ratings, and free-text entries. Array questions contained 5 to 10 features or topics, which the participants rated on a 4-point Likert scale (*translated from German:* not important at all, not important, important, and very important and not useful, minimally useful, useful, and very useful). An even-numbered scale was used to avoid central tendency bias. Free-text entries were conditionally displayed based on the preceding yes or no question; they allowed for expansion upon the chosen answer and additional comments. For each question, directions were provided to aid understanding (eg, “multiple answers may be chosen” and “please rate the following statements”). To ensure common understanding of the purpose of the proposed app, a narrative explanation of the app’s purpose and function was provided before any questions were presented. All questions except the free-text entries were mandatory for survey completion and submission. Participants were able to revise their answers using the forward and backward navigation buttons. Surveys were collected from August 2020 to December 2020.

### Statistical Analysis

Data from the anonymous surveys were analyzed using SPSS (version 27; IBM Corp) to analyze data in a primarily descriptive manner.

## Results

### Overview

Of the 213 unique survey visitors, 13 (6.1%) individuals visited the start page containing study information and informed consent but did not start the survey, and 35 (16.4%) individuals started the survey but did not complete it. The participation rate was 93.9% (200/213), and the completion rate was 77.5% (165/213). Only completed questionnaires (optional responses not required) were analyzed. Excluding 0.6% (1/165) of the surveys from a dentistry student, 99.4% (164/165) of the surveys were analyzed. Participant characteristics are summarized in Table 1. Generations were defined according to the divisions defined by the Pew Research Center [45].

**Table 1:** Participant characteristics (N=164).

Characteristics and subtypes		Participants, n (%)
**Sex**		
	Women	145 (88.4)
	Men	19 (11.6)
**Profession**		
	Physician (phoniatrician and ears, nose, and throat specialist)	32 (19.5)
	Speech-language pathologist	69 (42.1)
	Medical student (German: *Humanmedizin Studierende*)	20 (12.2)
	Speech-language pathology student	43 (26.2)
**Generation**		
	Generation Z (1996 and later)	56 (34.1)
	Generation Y or Millennials (1980-1995)	62 (37.8)
	Generation X (1965-1979)	33 (20.1)
	Baby Boomer (1946-1964)	13 (7.9)
**Professional experience (years)**		
	0^a^	60 (36.6)
	1-5	38 (23.2)
	6-10	15 (9.1)
	11-15	11 (6.7)
	16-20	18 (10.9)
	>20	22 (13.4)

^a^Still studying.

### General Interest in a *DLT* App

Of the 164 included participants, 162 (98.8%) participants expressed that they were open to trialing the proposed DLT app for subjects about CSD. In an optional follow-up response, 6.3% (2/32) physicians who indicated no interest in the proposed app cited concerns regarding app data collection. No significant differences across professions, years of experience, or generations were found. Regarding usefulness, 96.9% (159/164) of the participants reported that they found the proposed app helpful. In an optional follow-up free-response question, 3% (1/32) of the physicians indicated concerns about peer review of resources, and another 3% (1/32) of the physicians expressed that the topics were irrelevant to their current work. No significant differences across professions, generations, or years of experience were observed.

### App Functions

**Figure 1:**
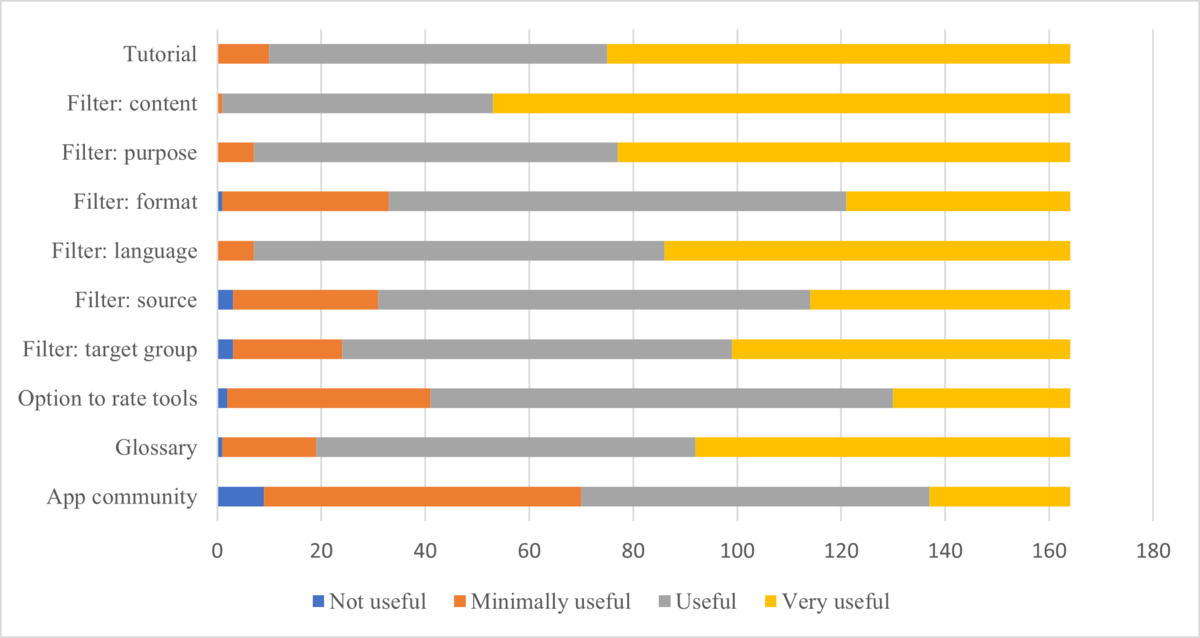
Perceived usefulness of selected app functions.

Participants were asked to rate selected app functions on a 4-point Likert scale (1=not useful, 2=minimally useful, 3=useful, and 4=very useful; translated from German). This scale was also used to rate app settings in the next section. Participants were asked to rate the following app functions: introductory tutorial; filter functions based on content, purpose, digital format, language, source, and target audience; tool rating function; glossary; and app community function. A summary of the perceived usefulness of these selected app functions is presented in Figure 1. Regarding the introductory tutorial, of the 164 participants, 89 (54.3%) participants rated the function as very useful, 65 (39.6%) rated it as useful, and 10 (6.1%) rated it as minimally useful. Of the 164 participants, 111 (67.7%) participants rated a filter function based on content or topic (eg, anatomy and specific disorder category) as very useful, 52 (31.7%) ranked it as useful, and 1 (0.6%) participant ranked it as minimally useful. Similarly, most participants found a filter function based on the purpose or focus of a digital tool (eg, general disorder overview and clinical measurement) as very useful (87/164, 53%) and useful (70/164, 42.7%), whereas 4.3% (7/164) of the participants rated the function to be minimally useful. Among the 164 participants, a filter function based on digital format was rated as very useful by 43 (26.2%) participants, useful by 88 (53.7%), minimally useful by 32 (19.5%), and not useful by 7 (4.3%) participants. Of the 164 participants, a filter function based on language was rated as very useful by 78 (47.6%) participants, useful by 79 (48.2%), and minimally useful by 7 (4.3%) participants. Of the 164 participants, 50 (30.5%) participants found a filter based on source (eg, digital tool created from a university vs commercial or industry) to be very useful, 83 (50.6%) found it to be useful, 28 (17.1%) found it to be minimally useful, and 3 (1.8%) participants found it to be not useful. Among the 164 participants, a filter function based on target audience (eg, students vs professionals) was rated as very useful by 65 (39.6%) participants to be very useful, useful by 75 (45.7%), minimally useful by 21 (12.8%), and not useful by 3 (1.8%) participants. Interestingly, although most participants found the option to rate tools as very useful (34/164, 20.7%) or useful (89/164, 54.3%), approximately one-fourth of the participants rated this as minimally (39/164, 23.8%) or not useful (2/164, 1.2%). Regarding the glossary function with digital learning and digital health terminology, of the 164 participants, 72 (43.9%) participants rated the function as very useful, 73 (44.5%) rated it as useful, 18 (10.9%) rated it as minimally useful, and 1 (0.6%) participant rated it as not useful. Finally, when rating the usefulness of an app community function, opinions varied greatly. Among the 164 participants, 27 (16.5%) participants rated the function as very useful, 67 (40.9%) rated it as useful, 61 (37.2%) rated it as minimally useful, and 9 (5.5%) rated it as not useful. Significant differences across professions were found regarding preference for an app community (*H_3_*=9.785; *P=*.02), specifically between physicians and medical students and between medical students and speech-language pathology students; however, pairwise comparisons were no longer significant when Bonferroni correction was applied.

When asked to provide additional desired functions in an optional free-response follow-up question, participants cited the challenges associated with specific functions and several interesting suggestions. An SLP cited a preference for text tutorials as they felt that video tutorials did not allow sufficient time for processing keywords or skipping irrelevant material. Additional functions suggested by SLPs included the incorporation of newsfeed feature, filter function based on complexity, text-to-speech function, examples of use, individualization options in settings, frequently asked questions (FAQs) function, and the ability to save certain content. Some of these features were presented in the question regarding app setting functions (eg, newsfeed feature and personalization options). Speech-language pathology students additionally suggested the option to track learning progress and link similar content to help with standardization or validation of certain digital tools. A physician also suggested the incorporation of audio or visual aids (eg, text-to-speech and larger font options) for students or professionals who need such supports.

### App Settings

**Figure 2:**
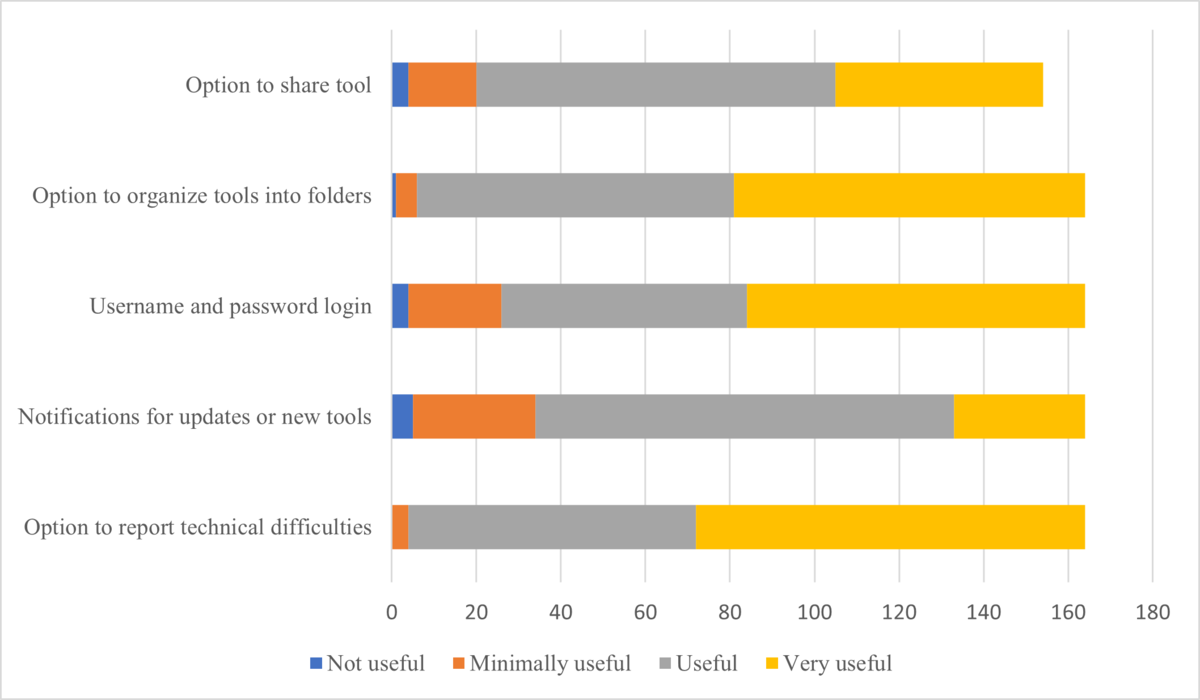
Perceived usefulness of selected setting functions.

Participants rated the app settings in terms of their perceived usefulness. Participants were specifically asked to rate the following app settings: option to share tools, option to organize tools into folders, username and password login, notifications for updates, and setting for reporting technical difficulties. A summary of the perceived usefulness of these app settings is shown in Figure 2. Regarding the option to share digital tools via social media links (eg, through email, WhatsApp, and Facebook), of the 164 participants, 49 (29.9%) participants rated such a function as very useful, 95 (57.9%) rated it as useful, 16 (9.8%) rated it as minimally useful, and 4 (2.4%) participants rated it as not useful. Of the 164 participants, 83 (50.6%) participants rated the settings option to organize and save tools into personalized categories and folders as very useful, 75 (45.7%) rated it as useful, 5 (3%) rated it as minimally useful, and 1 (0.6%) participant rated it as not useful. Regarding the incorporation of a username and password function, of the 164 participants, 80 (48.8%) participants found the setting to be very useful, 58 (35.4%) found it to be useful, 22 (13.4%) found it to be minimally useful, and 4 (2.4%) found it to be not useful. Significant differences were found across professional groups (*H*_3_=8.006; *P=*.046) regarding the username and password function; specifically, SLPs demonstrated greater preference for such a setting than their speech-language pathology student counterparts (*P*=.045) when Bonferroni correction was applied. Similarly, significant differences were found across generations (*H*_3_=9.309; *P=*.03), with Generation Z demonstrating significantly high distribution of opinions (*P*=.04), whereas Generation X primarily preferred a username and password function. Regarding a setting for receiving notifications for updates or the addition of new digital tools to the app library, of the 164 participants, 31 (18.9%) participants rated it as very useful, most participants (n=99, 60.4%) found it to be useful, 29 (17.7%) found it to be minimally useful, and 5 (3%) participants found it to be not useful. Of the 164 participants, most participants also agreed that a setting for reporting technical issues would be very useful (n=92, 56.1%) or useful (n=68, 41.5%), whereas 4 (2.4%) participants found it to be minimally useful. Significant differences were found across generations (*H_3_*=9.309; *P=*.02); however, these findings were no longer significant for pairwise comparisons when Bonferroni correction was applied.

When asked to provide additional desired functions in an optional free-response follow-up question, only SLPs and speech-language pathology students made additional comments. SLPs again emphasized the desire for a text-to-speech function and an FAQs section, whereas speech-language pathology students additionally suggested the option for data extraction to or synchronization with Microsoft Office or commonly used programs and the option to synchronize personalized digital libraries across multiple devices.

### Content Areas

**Figure 3:**
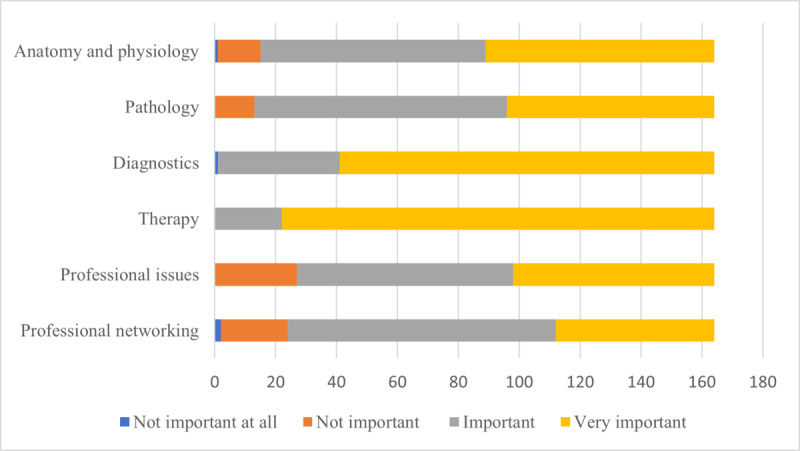
Perceived importance of selected clinical content areas.

Participants were asked to rate the perceived importance of various clinical and professional subjects on a 4-point Likert scale (1=not important at all, 2=not important, 3=important, and 4=very important). Participants were asked to rate the importance of the following content areas: anatomy and physiology, pathology, diagnostics, therapy, professional practice issues, and professional networking. A summary of the perceived importance of the selected clinical content areas is presented in Figure 3. Of the 164 participants, 75 (45.7%) participants found anatomy and physiology to be very important, 74 (45.1%) found them to be important, 14 (8.5%) found them to be not important, and 1 (0.6%) found them to be not important at all. Of the 164 participants, most participants rated the content area of pathology as very important (n=68, 41.5%) or important (n=83, 50.6%), whereas 13 (7.9%) participants found it to be not important. All except 1 participant found the subject of diagnostics to be either very important (123/164, 75%) or important (40/164, 24.4%), with significant difference across professional groups (*H*_3_=9.098; *P*=.03), specifically with greater variance in perceived importance of diagnostic topics among physicians than among SLPs (*P*=.02). All participants agreed that therapy was either a very important (142/164, 86.6%) or important content area (40/164, 24.4%); however, interestingly, significant differences were found across professional groups regarding perceived level of importance (*H*_3_=21.236; *P*<.001). In particular, physicians demonstrated significantly great variance regarding the perceived level of importance for topics related to therapy, whereas SLPs almost unanimously rated therapy as a very important content area (*P*=.02). Of the 164 participants, 66 (40.2%) participants rated professional issues as very important, 71 (43.3%) rated them as important, and 27 (16.5%) participants rated them as not important. Regarding the content area of professional networks, of the 164 participants, 52 (31.7%) participants perceived it to be very important, 88 (53.7%) perceived it to be important, 22 (13.4%) perceived it to be not important, and 2 (1.2%) participants perceived it to be not important at all.

### App Concerns

To gain insight into participants’ opinions regarding challenges or concerns regarding the proposed app, participants were asked to choose from suggested concerns and were also given the option of a free-response textbox to express their opinions. Although of the 164 participants, 53 (32.3%) participants indicated that they had no concerns at all, most (n=111, 67.7%) participants expressed concerns. Specifically, of the 164 participants, 69 (42.1%) participants expressed concerns about technical difficulties, 67 (40.9%) expressed concerns about data privacy and protection, and 13 (7.9%) participants expressed that they doubted the usefulness of such a proposed app. No significant differences across professions, generations, or years of experience were observed.

In the optional free-response textbox, SLPs expressed concerns regarding quality, the possibility of data overflow, and the need for keeping the app consistently updated. Speech-language pathology students indicated concerns regarding potential difficulties in knowing how to use all the app functions, the quality of the tools included in the library, and how digital tool ratings would be managed. In addition, physicians mentioned concerns about the long-term applicability of some of the tools included in the app library and the potential for limited exchange among users of the app. Notably, 2.4% (4/164) of the participants mentioned confusion and limited understanding of the proposed app and wanted to see a prototype to aid their evaluation.

## Discussion

### Overview

In this study, participants indicated readiness to trial a DLT app and reported desired functions that would increase utility and ability to share resources and help with knowledge management; they also reported concerns regarding technical barriers, data protection, quality, and sustainability.

Given the exponentially increasing number of digital resources that generates tremendous cognitive load, solutions for better management and evaluation of the vast amounts of incoming digital information are urgently needed. For professionals, lack of time and skills for effective searching has been previously shown to hamper effective integration of information into workflow and clinical practice. Moreover, speed of information access has been shown to take precedence over the quality of information, indicating a problematic lack of prioritization of evidence-based practice in the face of digital information overload [46-48]. For students, it has been demonstrated that providing access to information at the point of need promotes learner-centric knowledge and skill building, which is further facilitated through time management and access to resources other than those offered by their academic institutions [2,3,49,50]. Thus, to improve accessibility, quality, and manageability of digital resources and tools, this survey study sought to investigate the potential usefulness and desired features of a proposed collaborative library of digital tools and resources—a DLT app for CSD.

### General Interest in DLT App

The fact that most participants (162/164, 98.8%) indicated interest in trialing the proposed app is encouraging and demonstrates a readiness to engage in digital health topics, as seen in other studies with clinical professionals and students [6,11,23,34]. For the few participants who did not indicate interest in the app, their concerns of data collection are not unfounded. Given that many health apps are free and paid for, often using personal data for personalized advertisement and marketing, such fears are understandable [51]; however, it was not the intention of the proposed app to be used for any commercial purposes. Importantly, a participant indicated feeling that digital resources were not relevant to their current work. Such comments demonstrate that although most professionals and students may be open to digital developments, the degree of digitalization and acceptance varies. Moreover, there is a critical need for explicit digital skills and digital health education—both at the level of academic training and continuing professional development.

### App Functions

Regarding app functions, features (introductory tutorial, app rating function, glossary, and app community) and specific filter functions (based on content or topic, purpose, digital format, language, source, and target audience) were investigated. Given that introductory tutorials have been demonstrated to greatly increase the usability of an app, it is unsurprising that most survey participants rated this feature as very useful (89/164, 54.3%) or useful (65/164, 39.6%) [52]. However, notably, in an optional free-response follow-up, a participant mentioned the format of introductory tutorials, specifically pointing to the challenge of insufficient time for processing and understanding, given the increasing shift to video-based as opposed to only text-based tutorials. This aligns with previous literature demonstrating that although video-based tutorials often provided rich visual input and support, text-based introductory materials required less mental effort to comprehend and thus had an efficiency advantage [53]. Thus, although video tutorials organized by functions or settings could be useful for visual input, extraneous redundant processing could be reduced by eliminating unnecessary onscreen text, simple visuals, and the option to pause or skip sections [54].

Regarding tool or resource rating, it was notable that approximately one-fourth of the participants perceived the function to be minimally useful (39/164, 23.8%) or not useful (2/164, 1.2%). Considering the dire need for more quality assessment or peer-review processes for the increasing number of digital resources that continue to be unevaluated, it is interesting that high perceived importance was not found. Although it would be necessary to systematically explore and design the details of such an evaluation or rating function (eg, star ratings, commentaries, and standardized or nonstandardized scale ratings) for such a proposed app with experts, it has been suggested that formalized or standardized checklists, such as the Mobile App Rating Scale or the Interactive Mobile App Review Toolkit, could be used to improve quality assessment [55,56]. Systematically, well-designed review criteria could help professionals and students to have more tangible means of quickly attaining information regarding a digital tool’s evidence base, usefulness, problems, and costs among other factors [56]. However, in the long run, it will be important for professional regulating bodies to take a large part in regulating and promoting such review criteria to foster greater standardization of evaluation criteria [40,56].

Regarding glossary function, which defines common digital learning and digital health terminology, most participants rated the function as very useful (72/164, 43.9%) or useful (73/164, 44.5%). As demonstrated by the previously reported lack of confidence in digital health concepts, such a function could serve as an essential foundational reference and also be flexibly modified and adjusted as digitalization continues to advance [28,33-37]. Glossaries are crucial for building a basic common, agreed-upon understanding of specific concepts—which is critical in the ever-evolving digital environment, where new definitions and concepts are frequently emerging and must be adaptively adjusted [57].

Consistent with previous studies, overall, medical students showed more positive views toward an app community [28,58], and in this study more so than their professional counterparts, which, to the best of our knowledge, has not been previously reported. Such web-based learning environments could provide a space for problem solving and developing an interprofessional, collaborative community of team-based practice early in students’ academic and clinical careers [59]. For clinical educators, it has been suggested that digital communities in the form of *web-based communities of practice* could also serve as a valuable resource to exchange insights, build professional relationships, foster innovation, and generate successful scholarship of teaching and learning [59]. Moreover, such communities could additionally help to increase user engagement, thereby potentially helping to keep shared information and digital tools up-to-date and serving as a discussion platform for future app improvements. Nevertheless, there are reservations about the benefit of such a function, given that more than one-third of the study participants negatively rated the usefulness of an app community. Although it could be that participants simply prefer to engage in professional exchange elsewhere (eg, social media and existing platforms such as ResearchGate) [60,61], there is evidence that professionals and students are strategic and selective in their use of information-communication technology based on their perception of the extent to which certain tools will meet their operational needs, regardless of their level of digital skills [62,63]. Therefore, investigating the reasons or motivations for perceived utility in future studies would be insightful. In a web-based environment where the establishment of collegiality may be more difficult owing to limited direct social interaction, research has also demonstrated that supportive organizational culture, respect for cultural dimensions of exchange and intellectual insights, presence of personal knowledge–based trust, and availability of adequate exchange tools best foster openness to collaborative knowledge sharing and generation [62,64]. Moving forward, rigorous research into methods to craft such *intellectually safe* and connected web-based spaces most effectively will be critical.

Filter functions were also suggested to help facilitate the targeted identification of relevant tools. As previously mentioned, it has been suggested that the issue of information overload in our digital age may mostly be related to the ability of individuals to concretely use information at their disposal, which can be enhanced through information filters [38,39]. Although information filters based on content or topic (163/164, 99.4%), purpose (157/164, 95.7%), language (157/164, 95.7%), and target audience (140/164, 85.4%) were relatively common and agreed upon to be useful or very useful by most participants, filters for digital format type were considered to be minimally useful by 19.5% (32/164) or not useful by 4.3% (7/164) of the participants. This could be explained by the fact that clinical professionals and students may be more concerned with the content and value of the information itself as opposed to the format in which it is presented. Although incorporation of digital format type as a filter could be useful for identifying information that can be compatibly incorporated into academic or research presentations or even patient’s devices, it appears that basic knowledge of digital formats and digital literacy skills continues to be questionable and limited among clinical professionals and students, which again calls for the purposeful integration of health care–relevant digital skills training in academic and continuing education [33,65,66]. On the other hand, the source of a digital resource (eg, created by an academic institution or for commercial purposes), was considered to be very useful (50/164, 30.5%) or useful (83/164, 50.6%) by most participants. Given the currently low barrier for entering the app market, an increasing number of medical or clinical apps or digital resources are being developed by individuals or companies outside the health care sector. Although some have engaged expert opinion, others have not and often do not have the clinical insights to ensure the scientific or clinical quality of the resource, unlike academic institutions where quality evaluation of evidence base may be easier to ensure; however, they may not always be effective [67,68]. Here, it is also useful to mention that under current entrepreneurial models, the creation of *minimum viable products* including for medical apps or digital tools are introduced to the market with the intention of gradual improvement based on user feedback over time. This means that immature or nonoptimized resources, whether for patient or educational use, are being used when their quality or efficacy has not yet been established [69]. This highlights a great challenge of understanding how to shift strategies for evidence-based practices in the context of rapid digital progression—although a filter function based on source will not help to tackle this complex question, it may serve as a first step to encourage professionals and students to be more critical of the resources they choose to use.

### App Settings

App setting options were explored to determine personalization functions that could help to enhance the usability of the proposed app. Consistent with findings that suggest that students and clinical professionals are open to and use social media for academic and clinical purposes, most participants (144/164, 87.8%) positively rated the option to share tools via social media [12,70,71]. Importantly, although it has been demonstrated that the use of social media for the sharing of clinical information can be helpful for quick and easy dissemination, it is notable that social networks serve as information filters that may rather highlight digital resources of personal popularity as opposed to true clinical evidence base and quality [38]. Moreover, social media as a digital means of clinical learning has demonstrated questionable educational value and unclear evidence regarding its effect on learning and performance outcomes [70,71].

Regarding personalization functions, an overwhelming majority of the survey participants positively rated the option to organize and save tools into folders. Such a function could serve as an additional layer of information filtering and support the encoding, organization, and synthesis of data as professionals and students try to understand the digital resources and tools that they find more relevant [54]. Therefore, it was encouraging to see the suggestion by several individuals for the option for linking similar content and for extraction to commonly used programs such as Microsoft Office. The suggestion of compatibility options across multiple devices further supported clinicians’ and students’ desires for easy accessibility, which could potentially increase the uptake of the proposed app. As previously demonstrated, technical software issues including those associated with nontransferability and noncompatibility across different mobile devices were barriers to learning efficacy; individuals were more likely to implement solutions that increased accessibility [5]. The additional suggestion of audiovisual aids (eg, text-to-speech and larger font options) for users with specific needs perhaps reflects the field’s general focus on disability supports, given the clinical populations that are typically affected by communication disorders [72].

Regarding personalization of functions and features, the suggestion of a username and password function—which would likely be needed to save such settings—was met with significant variance across professional groups and generations. Specifically, SLPs indicated greater preference in comparison with their speech-language pathology student counterparts, as did Generation X in comparison with Generation Z. Regarding generational differences, although it is particularly difficult to delineate why professional group differences were found among SLPs and speech-language pathology students, it has been previously found that middle-aged individuals (aged 45-60 years, belonging to Generation X) had more negative views regarding data disclosure than their younger counterparts (aged 19-24 years, belonging to Generation Z) and may thus demonstrate greater preference toward password protections [73].

Most participants (130/164, 79.3%) also positively rated notifications for updates and new digital resources and the setting for reporting technical issues. Importantly, notifications, which were also requested by a survey participant in the form of *newsfeed* feature, have been associated with both benefits and drawbacks. Although notifications could help to keep users up-to-date and readily informed, they can be disruptive or further contribute to information overload [74]. Thus, it is important to design setting functions that allow the user to manage or turn off push notifications or to incorporate updates as a newsfeed feature only. Furthermore, the incorporation of a function for reporting technical difficulties has been identified as a critical criterion for app development, maintenance, and improvement [75].

### Content Areas

It has been suggested that solutions for information overload can involve, among technological solutions and improved digital literacy, the creation or adaptation of specific content [38]. Subject-focused materials have been demonstrated to improve timely access to relevant material, which in turn can benefit the storage and retrieval of learned information [76]. Thus, perceptions about specific content filters were also explored. The foundational content areas of anatomy and physiology and pathology were rated by most participants to be useful, regardless of their profession, generation, or years of experience. However, physicians demonstrated significantly greater variance in the perceived importance of diagnostics and therapy than their speech-language pathology counterparts, who provided overwhelmingly positive ratings for both topics. Although it is more difficult to explain the significant differences observed in terms of perceived importance of diagnostic content (clinical responsibilities that fall within both physicians’ and SLPs’ responsibilities, although different in specific scope), differences in the perceived importance of therapy content can potentially be explained by the fact that SLPs take a large part in therapy in their scope of practice [77]. Furthermore, although professional issues and networking may more directly affect working clinical professionals, this study found no significant differences in the perceived importance of such topics among participants across professional groups, generations, or years of experience. This is promising as it suggests that students also value the professional skills and networks that will be crucial to their future work. However, importantly, evidence suggests that such perceived value may not necessarily translate to effective practice without appropriate guidance or explicit teaching [78].

### App Concerns

It is well reported that there are clinical professional and student concerns about the quality of digital resources for both patient and academic purposes [40,79,80]. Thus, the concerns of the survey participants were investigated to better understand how to craft more effective future solutions. Although approximately one-third of the participants (53/164, 32.3%) reported no concerns with the suggested app at all, most participants (111/164, 67.7%) highlighted concerns about technical difficulties, data privacy and protection, and utility of the suggested app and several self-reported concerns in the optional free-response follow-up question. Technical difficulties can potentially be addressed through the proposed introductory tutorial, technical difficulty reporting function, or even the participant-suggested FAQs section, which can be a basic troubleshooting page. Data privacy concerns are well reported among health-related apps and digital tools, especially considering the vast amounts of sensitive health-related and personal information that can be collected through such means [81]. Although it was not the intention of the proposed app—which serves as a reference tool as opposed to an app for medical purposes or diagnostics—to collect any personal data other than potential username and password information for app personalization, this concern highlights the importance of ensuring that all apps and digital resource strictly align with current legal frameworks (eg, General Data Protection Regulation) to protect sensitive personal or medical data [82,83]. In addition, it is useful to mention that the concern of reduced exchange and interaction could be addressed through the incorporation of the suggested app community function; however, this would inevitably increase the amount of personal data that would need to be saved or anonymous aliases could be used. Nevertheless, it is worth re-emphasizing that the primary purpose of the proposed app was first and foremost to serve as a collaborative digital library of digital resources for CSD.

Another concern that necessitates further discussion is the need for consistent updates and app maintenance. Lack of app maintenance is reportedly a major reason for the failure of many apps [84]. Thus, to help facilitate the viability of the proposed digital library and reference app, it could be useful to include the option to suggest tools as a part of a collaborative effort to keep the included digital resources and tools up-to-date. It has also been previously suggested that a *curated app repository* that includes apps meeting minimum standards could be managed through *risk-based app triage*, which could be partially automated based on criteria such as the previously mentioned quality checklists (eg, Mobile App Rating Scale and Interactive Mobile App Review Toolkit). Thresholds can be set for determining when apps pose low risk and can undergo a more automated evaluation process, whereas other aspects could be more thoroughly and manually evaluated [40]. Such a system can potentially be applied to the proposed digital tool library that includes resources beyond just mobile apps. Furthermore, as mentioned by a survey participant, it is also important to consider the long-term relevance of digital resources or when a resource should be rendered obsolete. As mentioned previously, in the current age of continuously incoming data, the management of such information requires constant strategizing. Resources can quickly become irrelevant and, for reasons of limited data storage capacity, would have to be removed. To address this challenge, digital resources and tools can similarly undergo the previously mentioned triage process, with a shifted focus toward the timeliness and relevance of the digital resource.

### Limitations

Although this study has demonstrated promising interest in the proposed digital reference app for CSD professionals and students, it must be considered with its limitations in mind. First, this study investigated perceptions toward a proposed app based on a narrative description. Although only 2.4% (4/164) of the participants indicated difficulty in understanding the intentions of the app as no prototype was offered as an objective reference, their understanding of the proposed app could have varied and informed their reported perceptions. The decision to implement a questionnaire before the prototype was made in light of previous literature citing lack of knowledge of users’ demands and expectations as a key reason for prototype failure [84,85]. Thus, we deliberately chose to implement a co-design approach in which stakeholder insights were incorporated from the forefront to determine whether such a digital app would be desirable and inquired into need areas and preferences that could be used to ideally support more sustainable future development [86,87]. Regarding external validity, it is important to emphasize that the study was conducted in German-speaking countries only, and thus, perceptions and attitudes may likely differ from other cultural or geographical contexts. However, given the global reach of digitalization and the rather comprehensive nature of the proposed digital library reference app, the study findings could help to highlight common or global trends and useful resources across the field of CSD internationally. It is also useful to mention that, as an open survey, a convenience sample was collected; thus, it is possible that individuals who already held greater interest in digital topics were more likely to participate in the survey. Notably, many of the survey participants had ≤5 years of professional experience, which could have certainly affected their perceptions and attitudes toward such an app. In future studies, it would be useful to investigate whether more experienced professionals would demonstrate different preferences or insights into the utility of such a digital resource. Similarly, given this convenience sample, it was difficult to account for differences in group sizes; however, statistical adjustments to data analyses were made as appropriate. The great disparity between male and female participants further highlights the larger trend of a rather female-dominated field of speech-language pathology and the increasing number of women entering the medical specialty of otolaryngology [88,89]. In light of the exponential pace of digital progress, this survey study reveals the current needs, preferences, and perceptions regarding a proposed digital reference tool, which will likely evolve to include other needed supports as new digital resources, formats, and challenges arise.

### Future Directions

As this study has only started to explore the potential utility of a collaborative and interdisciplinary digital library reference app, moving forward, it will be critical to further investigate, design, and test desired settings and functions to determine whether stakeholders’ perceptions align with the actual use and implementation of such a tool. Even after prototype creation, several rounds of stakeholder evaluation and testing would be preferable to ensure that such an app is not released prematurely without proper initial evidence. Nevertheless, ongoing re-evaluation and improvement would be necessary given the ever-changing digital health care environment [68,90]. As digital learning and health apps continue to emerge, it will be critical that resources are tailored to specific target audiences, these stakeholders are engaged in digital resource creation and evaluation processes, and ongoing technical assistance is explicitly integrated into support tools [5]. As current knowledge and awareness of the range of available digital resources in the interdisciplinary field of CSD is limited, approaching the challenge with a digital *library* or *repository* can help to increase awareness, access, management, sharing, and, ideally, future quality assessment of available and emerging digital resources [90]. Such a learning reference tool could additionally serve as an important prerequisite for investigation into digital tools for clinical use. In the future, investigating the utility of such an app for those working in other related interdisciplinary fields, such as occupational therapists or nurses working in CSD, could also be useful.

Our study has demonstrated that clinical professionals and students in CSD are open to trialing a repository-like, collaborative, interdisciplinary digital library reference app and prefer features and functions that optimize usability, allow personalization, and increase exchanges regarding the quality assessment and evidence base for digital resources and tools. These stakeholders prefer a wide range of content topics and have reasonable concerns about the technical or data privacy challenges associated with app use; however, they are ready to explore new solutions for more efficient and effective knowledge and information management. The digital age is presenting opportunities and challenges for clinical teaching, learning, and practice that “...result in a richer range of resources to support practice and learning, but also creates conflicting evidence, insecurity about the knowledge and greater demands on the professional to identify the appropriate knowledge for their problem in question” [91]. As the digital health care landscape continues to advance at an unpredictable pace, information overload will be inevitable and will require traditional means of collecting, managing, and evaluating clinical information to adaptively evolve through ongoing cycles of evaluation to focus on and improve clinical decision-making, research, and clinical practice.

## Appendix

Multimedia Appendix 1 Survey screens (in German).

## Abbreviations

CHERRIES: Checklist for Reporting Results of Internet E-Surveys
CSD: communication sciences and disorders
DLT: digital learning toolbox
FAQ: frequently asked question
PROMIS: Patient-Reported Outcomes Measurement Information System
SLP: speech-language pathologist

## References

[1] Ellaway R, Masters K (2008). AMEE Guide 32: e-Learning in medical education Part 1: learning, teaching and assessment. Med Teach.

[2] Davies BS, Rafique J, Vincent TR, Fairclough J, Packer MH, Vincent R, Haq I (2012). Mobile Medical Education (MoMEd) - how mobile information resources contribute to learning for undergraduate clinical students - a mixed methods study. BMC Med Educ.

[3] Wallace S, Clark M, White J (2012). 'It's on my iPhone': attitudes to the use of mobile computing devices in medical education, a mixed-methods study. BMJ Open.

[4] Ozdalga E, Ozdalga A, Ahuja N (2012). The smartphone in medicine: a review of current and potential use among physicians and students. J Med Internet Res.

[5] Green BL, Kennedy I, Hassanzadeh H, Sharma S, Frith G, Darling JC (2015). A semi-quantitative and thematic analysis of medical student attitudes towards M-Learning. J Eval Clin Pract.

[6] Nguyen L, Barton SM, Nguyen LT (2014). iPads in higher education-hype and hope. Br J Educ Technol.

[7] Goh P, Sandars J (2020). A vision of the use of technology in medical education after the COVID-19 pandemic. MedEdPublish.

[8] Iwai Y (2020). Online learning during the COVID-19 pandemic. Scientific American Blog Network.

[9] Ahmed H, Allaf M, Elghazaly H (2020). COVID-19 and medical education. Lancet Infect Dis.

[10] (2011). mHealth: new horizons for health through mobile technologies. World Health Organization.

[11] Dunleavy G, Nikolaou CK, Nifakos S, Atun R, Law GC, Tudor Car L (2019). Mobile digital education for health professions: systematic review and meta-analysis by the digital health education collaboration. J Med Internet Res.

[12] Bahner DP, Adkins E, Patel N, Donley C, Nagel R, Kman NE (2012). How we use social media to supplement a novel curriculum in medical education. Med Teach.

[13] Chase TJ, Julius A, Chandan JS, Powell E, Hall CS, Phillips BL, Burnett R, Gill D, Fernando B (2018). Mobile learning in medicine: an evaluation of attitudes and behaviours of medical students. BMC Med Educ.

[14] Nerminathan A, Harrison A, Phelps M, Alexander S, Scott KM (2017). Doctors' use of mobile devices in the clinical setting: a mixed methods study. Intern Med J.

[15] Chang CY, Hwang GJ (2018). Trends of mobile technology-enhanced medical education: a review of journal publications from 1998 to 2016. Int J Mob Learn Organ.

[16] Thompson K, Zimmerman E (2019). Pediatric speech-language pathologists' use of mobile health technology: qualitative questionnaire study. JMIR Rehabil Assist Technol.

[17] (2020). Disability and health. World Health Organization.

[18] Ahmadvand A, Gatchel R, Brownstein J, Nissen L (2018). The biopsychosocial-digital approach to health and disease: call for a paradigm expansion. J Med Internet Res.

[19] Nelson R, Staggers N (2016). Health informatics: an interprofessional approach.

[20] Kurpinski K, Johnson T, Kumar S, Desai T, Li S (2014). Mastering translational medicine: interdisciplinary education for a new generation. Sci Transl Med.

[21] Hollweg W, Beck EM, Schulenburg K, Trock S, Räbiger J, Kraus E, Borde T (2016). Interprofessional health care - field of study with future and challenges / Interprofessionelle Versorgung – Ein Studiengebiet mit Zukunft und Herausforderungen. Int J Health Prof.

[22] Theodoros D (2012). A new era in speech-language pathology practice: innovation and diversification. Int J Speech Lang Pathol.

[23] Andrews T, Davidson B, Hill A, Sloane D, Woodhouse L, Kitchenham A (2011). Using students’ own mobile technologies to support clinical competency development in speech pathology. Models for Interdisciplinary Mobile Learning: Delivering Information to Students.

[24] Edwards J, Dukhovny E (2017). Technology training in speech-language pathology: a focus on tablets and apps. Perspect ASHA Spec Interest Groups.

[25] Leinweber J, Dockweiler C (2020). Perspektiven der Digitalisierung in der Logopädie/Sprachtherapie. forum:logopädie.

[26] Kuperstock JE, Horný M, Platt MP (2019). Mobile app technology is associated with improved otolaryngology resident in-service performance. Laryngoscope.

[27] Hsueh WD, Bent JP, Moskowitz HS (2018). An app to enhance resident education in otolaryngology. Laryngoscope.

[28] Tarpada SP, Hsueh WD, Gibber MJ (2017). Resident and student education in otolaryngology: a 10-year update on e-learning. Laryngoscope.

[29] Rak K, Völker J, Taeger J, Bahmer A, Hagen R, Albrecht UV (2019). Medical apps in oto-rhino-laryngology. Laryngorhinootologie.

[30] Hodes RJ, Insel TR, Landis SC, NIH Blueprint for Neuroscience Research (2013). The NIH toolbox: setting a standard for biomedical research. Neurology.

[31] Ader DN (2007). Developing the patient-reported outcomes measurement information system (PROMIS). Med Care.

[32] Torous J, Vaidyam A (2020). Multiple uses of app instead of using multiple apps - a case for rethinking the digital health technology toolbox. Epidemiol Psychiatr Sci.

[33] Lam MK, Hines M, Lowe R, Nagarajan S, Keep M, Penman M, Power E (2016). Preparedness for eHealth: health sciences students' knowledge, skills, and confidence. J Inf Technol.

[34] Machleid F, Kaczmarczyk R, Johann D, Balčiūnas J, Atienza-Carbonell B, von Maltzahn F, Mosch L (2020). Perceptions of digital health education among European medical students: mixed methods survey. J Med Internet Res.

[35] (2020). Recommendations by the next generation. European Health Parliament.

[36] Gagnon MP, Ngangue P, Payne-Gagnon J, Desmartis M (2016). m-Health adoption by healthcare professionals: a systematic review. J Am Med Inform Assoc.

[37] Alwani M, Bandali E, Larsen M, Shipchandler TZ, Ting J (2019). Current state of surgical simulation training in otolaryngology: systematic review of simulation training models. Arch Otolaryngol Head Neck Surg.

[38] Klerings I, Weinhandl AS, Thaler KJ (2015). Information overload in healthcare: too much of a good thing?. Z Evid Fortbild Qual Gesundhwes.

[39] Shirky C (2008). It’s not information overload. It’s filter failure. MAS Context.

[40] Wyatt JC (2018). How can clinicians, specialty societies and others evaluate and improve the quality of apps for patient use?. BMC Med.

[41] Boruff JT, Storie D (2014). Mobile devices in medicine: a survey of how medical students, residents, and faculty use smartphones and other mobile devices to find information. J Med Libr Assoc.

[42] Eysenbach G (2004). Improving the quality of Web surveys: the checklist for reporting results of internet E-surveys (CHERRIES). J Med Internet Res.

[43] Lin Y, Lemos M, Neuschaefer-Rube C (2021). Digital health and digital learning experiences across speech-language pathology, phoniatrics, and otolaryngology: interdisciplinary survey study. JMIR Med Educ.

[44] Schmitz C (2020). What you need to know about data security in LimeSurvey. LimeSurvey.

[45] Dimock M (2019). Defining generations: where Millennials end and Generation Z begins. Pew Research Center.

[46] Clarke MA, Belden JL, Koopman RJ, Steege LM, Moore JL, Canfield SM, Kim MS (2013). Information needs and information-seeking behaviour analysis of primary care physicians and nurses: a literature review. Health Info Libr J.

[47] Fox S (2013). After Dr Google: peer-to-peer health care. Pediatrics.

[48] Duran-Nelson A, Gladding S, Beattie J, Nixon LJ (2013). Should we Google it? Resource use by internal medicine residents for point-of-care clinical decision making. Acad Med.

[49] Spector JM, Anderson TM (2000). Integrated and holistic perspectives on learning, instruction and technology: understanding complexity.

[50] Fuller R, Joynes V (2014). Should mobile learning be compulsory for preparing students for learning in the workplace?. Br J Educ Technol.

[51] Huckvale K, Prieto JT, Tilney M, Benghozi PJ, Car J (2015). Unaddressed privacy risks in accredited health and wellness apps: a cross-sectional systematic assessment. BMC Med.

[52] Xie B, Shabir I, Abelson H (2015). Measuring the usability and capability of app inventor to create mobile applications. Proceedings of the 3rd International Workshop on Programming for Mobile and Touch.

[53] Hefter MH, Berthold K (2020). Preparing learners to self-explain video examples: text or video introduction?. Comput Human Behav.

[54] Clark RC, Mayer RE (2016). e-Learning and the science of instruction: proven guidelines for consumers and designers of multimedia learning. 4th edition.

[55] Stoyanov SR, Hides L, Kavanagh DJ, Zelenko O, Tjondronegoro D, Mani M (2015). Mobile app rating scale: a new tool for assessing the quality of health mobile apps. JMIR Mhealth Uhealth.

[56] Maheu MM, Nicolucci V, Pulier ML, Wall KM, Frye TJ, Hudlicka E (2016). The interactive mobile app review toolkit (IMART): a clinical practice-oriented system. J Technol Behav Sci.

[57] Holland PG (2002). The importance of glossaries. Flow Meas Instrum.

[58] Mehta NB, Hull AL, Young JB, Stoller JK (2013). Just imagine: new paradigms for medical education. Acad Med.

[59] Yarris LM, Chan TM, Gottlieb M, Juve AM (2019). Finding your people in the digital age: virtual communities of practice to promote education scholarship. J Grad Med Educ.

[60] Curran V, Matthews L, Fleet L, Simmons K, Gustafson DL, Wetsch L (2017). A review of digital, social, and mobile technologies in health professional education. J Contin Educ Health Prof.

[61] Manca S (2018). ResearchGate and Academia.edu as networked socio-technical systems for scholarly communication: a literature review. Res Learn Technol.

[62] Gannon-Leary P, Fontainha E (2007). Communities of practice and virtual learning communities: benefits, barriers and success factors. eLearning Papers.

[63] Smith PJ, Barty K, Stacey EA (2005). Limitations of an established community of practice in developing online innovation. CiteSeerx.

[64] Ardichvili A (2008). Learning and knowledge sharing in virtual communities of practice: motivators, barriers, and enablers. Adv Dev Hum Resour.

[65] Dattakumar A, Gray K, Henderson KB, Maeder A, Chenery H (2012). We are not educating the future clinical health professional workforce adequately for e-health competence: findings of an Australian study. Stud Health Technol Inform.

[66] Konttila J, Siira H, Kyngäs H, Lahtinen M, Elo S, Kääriäinen M, Kaakinen P, Oikarinen A, Yamakawa M, Fukui S, Utsumi M, Higami Y, Higuchi A, Mikkonen K (2019). Healthcare professionals' competence in digitalisation: a systematic review. J Clin Nurs.

[67] Grundy QH, Wang Z, Bero LA (2016). Challenges in assessing mobile health app quality: a systematic review of prevalent and innovative methods. Am J Prev Med.

[68] Price L, Kirkwood A (2013). Using technology for teaching and learning in higher education: a critical review of the role of evidence in informing practice. High Educ Res Dev.

[69] (2015). What is a minimum viable product (MVP)?. Entrepreneur Handbook.

[70] Sterling M, Leung P, Wright D, Bishop TF (2017). The use of social media in graduate medical education: a systematic review. Acad Med.

[71] Sutherland S, Jalali A (2017). Social media as an open-learning resource in medical education: current perspectives. Adv Med Educ Pract.

[72] Gillam RB, Marquardt TP (2019). Communication sciences and disorders: from science to clinical practice. 4th edition.

[73] Miltgen CL, Peyrat-Guillard D (2014). Cultural and generational influences on privacy concerns: a qualitative study in seven European countries. Eur J Inf Syst.

[74] Westermann T, Möller S, Wechsung I (2015). Assessing the relationship between technical affinity, stress and notifications on smartphones. Proceedings of the 17th International Conference on Human-Computer Interaction with Mobile Devices and Services Adjunct.

[75] Llorens-Vernet P, Miró J (2020). Standards for mobile health-related apps: systematic review and development of a guide. JMIR Mhealth Uhealth.

[76] Swanwick T, Swanwick T (2013). Understanding medical education. Understanding medical education: evidence, theory and practice. 2nd ed.

[77] (2016). Scope of practice in speech-language pathology. American Speech-Language-Hearing Association (ASHA).

[78] Cruess RL, Cruess SR, Steinert Y (2016). Teaching medical professionalism: supporting the development of a professional identity.

[79] Boulos MN, Brewer AC, Karimkhani C, Buller DB, Dellavalle RP (2014). Mobile medical and health apps: state of the art, concerns, regulatory control and certification. Online J Public Health Inform.

[80] McMillan B, Hickey E, Mitchell C, Patel M (2015). The need for quality assurance of health apps. BMJ.

[81] Benjumea J, Ropero J, Rivera-Romero O, Dorronzoro-Zubiete E, Carrasco A (2020). Privacy assessment in mobile health apps: scoping review. JMIR Mhealth Uhealth.

[82] Thorarensen B (2017). The processing of health information- protecting the individual right to privacy through effective legal remedies. Health Technol.

[83] Sokolovska A, Kocarev L (2018). Integrating technical and legal concepts of privacy. IEEE Access.

[84] N Inukollu V, Keshamon DD, Kang T, Inukollu M (2014). Factors influencing quality of mobile apps: role of mobile app development life cycle. Int J Softw Eng Appl.

[85] Deininger M, Daly SR, Lee JC, Seifert CM, Sienko KH (2019). Prototyping for context: exploring stakeholder feedback based on prototype type, stakeholder group and question type. Res Eng Des.

[86] Sandholzer M, Rurik I, Deutsch T, Frese T (2014). Medical students' expectations towards an implementation of a family medicine textbook as a comprehensive app in Germany. J Med Syst.

[87] Kildea J, Battista J, Cabral B, Hendren L, Herrera D, Hijal T, Joseph A (2019). Design and development of a person-centered patient portal using participatory stakeholder co-design. J Med Internet Res.

[88] (2019). Logopädie/Sprachtherapie: Schluss mit der Benachteiligung der Frauenberufe!. Deutscher Bundesverband für Logopädie (DBL).

[89] (2020). Geschlecht. Kassenärztliche Bundesvereinigung (KBV).

[90] Sousa MJ, Rocha Á (2019). Strategic knowledge management in the digital age: JBR special issue editorial. J Bus Res.

[91] Bullock A (2014). Does technology help doctors to access, use and share knowledge?. Med Educ.

