# Supplementary material for: Digital Health and Learning in Speech-Language Pathology, Phoniatrics, and Otolaryngology: Survey Study for Designing a Digital Learning Toolbox App
Source: JMIR Med Educ. 2022 Apr 27;8(2):e34042. doi: 10.2196/34042 (PMC9096631; doi:10.2196/34042)
Supplement: Multimedia Appendix 1 [file mededu_v8i2e34042_app1.pdf]

Multimedia Appendix 1. Survey Screens (*in German*)

**Sections of the introduction pages containing personal and/or contact information of faculty members have been removed for privacy**

# Knowledge and Attitudes Towards Digital Health and Learning in Speech-Language Pathology and Phoniatrics/ENT Medicine: A Survey for Designing a Digital Learning Toolbox

Sehr geehrte Probandin / Sehr geehrter Proband,

vielen Dank für Ihr Interesse an unserer Studie. Wir möchten Sie im Folgenden über die Studie informieren und offene Fragen mit Ihnen klären.

**Hintergrund:** Das digitale Zeitalter hat der medizinischen Lehre und Praxis neue Möglichkeiten und Herausforderungen eröffnet. Daher ist es für uns wichtig, mehr Einblick in Ihre Kenntnisse, Erfahrungen und Meinungen in Bezug auf digitale Gesundheit, digitales Lernen und aktuelle digitale Lern- und Therapietools zu erhalten. Diese Informationen werden uns helfen, digitale Bedürfnisse und Präferenzen zu identifizieren. Diese sollen dann zur besseren Bewertung und Optimierung aktueller und zukünftiger Tools beitragen und somit unsere Bereiche besser auf die sich digitalisierende Zukunft vorbereiten.

**Das Ziel** dieses Projektes ist es, Ihr Nutzungsverhalten, Ihre Erfahrungen, Bedürfnisse und Einstellungen zu digitalen Lern- und Therapietools zu untersuchen und die Nützlichkeit einer hypothetischen "Digital Learning Toolbox" -App sowie deren Merkmale und Funktionen zu ermitteln. Ihre Antworten und angegebenen Präferenzen würden uns dabei unterstützen, ein zukünftiges digitales Tool, das für unsere Bereiche Logopädie und Phoniatrie / HNO-Heilkunde geeignet ist, besser zu planen, zu strukturieren und aufzubauen.

**Wer kann teilnehmen?**

Die Umfrage richtet sich an Logopäden*innen, Phoniater*innen, HNO-Ärzte*innen, die zur Zeit arbeiten, sowie Studierende der Medizin oder Logopädie.

**Datenverarbeitung und Datenschutz**

Die Online Befragung erfolgt mit LimeSurvey des Audiovisuellen Medienzentrums (AVMZ) der Medizinischen Fakultät der RWTH Aachen und ist anonym. Im Rahmen der Studie werden Ihre Daten anonym gespeichert, wissenschaftlich ausgewertet und den gesetzlichen Bestimmungen entsprechend archiviert. Es werden keine Informationen erhoben, die Rückschlüsse auf Ihre Identität ermöglichen. Ihr Name, Ihre Adresse und Ihre IP-Adresse werden nicht erfasst. Es werden lediglich Ihr Alter, Ihr Geschlecht und Ihr Beruf erhoben. Eine Mehrfachteilnahme wird durch das Setzen von Cookies verhindert. Die Datenschutzbestimmungen von LimeSurvey.org finden Sie unter: https://www.limesurvey.org/de/data-protection-statement

Jedwede Veröffentlichung von Forschungsergebnissen erfolgt in anonymer Form. Damit ist gewährleistet, dass hierüber Rückschlüsse auf Sie als Person unmöglich sind. Die Beachtung des Bundesdatenschutzgesetztes ist in vollem Umfang sichergestellt.

**Freiwilligkeit**

Ihre Einwilligung, an diesem Forschungsvorhaben als Proband*in teilzunehmen, erfolgt ganz und gar freiwillig. Da Daten anonymisiert werden, können Sie Ihre Einwilligung nicht zurückziehen.

Falls Sie Rückfragen haben oder nähere Informationen wünschen, stehen wir Ihnen sehr gerne zur Verfügung.

Dies ist eine anonyme Umfrage.

In den Umfrageantworten werden keine persönlichen Informationen über Sie gespeichert, es sei denn, in einer Frage wird explizit danach gefragt.

Wenn Sie für diese Umfrage einen Zugangscode benutzt haben, so können Sie sicher sein, dass der Zugangsschlüssel nicht zusammen mit den Daten abgespeichert wurde. Er wird in einer getrennten Tabelle aufbewahrt und nur aktualisiert, um zu speichern, ob Sie diese Umfrage abgeschlossen haben oder nicht. Es gibt keinen Weg, die Zugangscodes mit den Umfrageergebnissen zusammenzuführen.

Ich erkläre mich damit einverstanden, an der Studie „Knowledge and Attitudes Towards Digital Health and Learning in Speech-Language Pathology and Phoniatrics/ENT Medicine: A Survey for Designing a Digital Learning Toolbox" teilzunehmen. Ich habe die Probandeninformation und die Datenschutzerklärung gelesen und fühle mich ausreichend informiert und habe verstanden, worum es geht. Meine Einwilligung, an diesem Forschungsvorhaben als Proband*in teilzunehmen, erfolgt ganz und gar freiwillig. Da die Daten anonymisiert werden, verstehe ich, dass ich meine Einwilligung nicht zurückziehen kann, da weder ich noch meine Daten nachträglich identifizierbar sind.

**Einverstanden** ☑

Weiter

***English narrative explanation of proposed app's purpose**:
*The goal is to understand the usefulness of a proposed "Digital Learning Toolbox" App and desired features and functions. Your responses and the preferences you report will help us to better plan, structure, and develop a future digital tool specifically catered to the needs of our fields of speech-language pathology and phoniatrics/otolaryngology.*

# Soziodemografische Informationen

*In welchem Beruf arbeiten Sie zurzeit?

Bitte wählen Sie eine der folgenden Antworten:

- Arzt/Ärztin (Phoniatrie/HNO)
- Logopäde/Logopädin oder akademische(r) Sprachtherapeut/Sprachtherapeutin
- Studierende der Logopädie/Sprachtherapie
- Studierende der Humanmedizin
- Sonstiges: ______

*Wie lange arbeiten Sie bereits in diesem Beruf?

Bitte wählen Sie eine der folgenden Antworten:

- Ich studiere noch
- 1-5 Jahre
- 6-10 Jahre
- 11-15 Jahre
- 16-20 Jahre
- Mehr als 20 Jahre

*Welcher Generation gehören Sie an?

Bitte wählen Sie eine der folgenden Antworten:

- Generation Z (Geburtsjahre ab 1996)
- Generation Y Millennials (Geburtsjahre 1980 - 1995)
- Generation X (Geburtsjahre 1965 - 1979)
- Babyboomer (Geburtsjahre 1946 - 1964)

*Geschlecht:

Bitte wählen Sie eine der folgenden Antworten:

- männlich
- weiblich
- divers

Zurück

Weiter

ZWEITER TEIL: App-relevante Fragen 1

***Im folgenden Abschnitt stellen wir Fragen zu einer hypothetischen "Digital Tool Box" App. Der beabsichtigte Zweck dieser App wäre es, klinischen Fachleuten und Studierenden Zugang zu einer Bibliothek von Open-Source-Tools für digitales Lernen und digitale Therapie zu verschaffen.*** *Dies könnte zu weiteren Erkenntnissen hinsichtlich digitaler Tools in unseren interdisziplinären Bereichen, auch in Bezug auf deren Qualitätsbewertung, beitragen.*

*Wären Sie generell bereit, eine App für unsere interdisziplinären Bereiche Phoniatrie / HNO und Logopädie zu nutzen oder auszuprobieren?

Ja | Nein

Warum würden Sie eine App nicht ausprobieren wollen?

*Würden Sie im Allgemeinen eine App, wie die zuvor beschriebene als hilfreich empfinden?

Ja | Nein

Warum würden Sie eine solche App nicht hilfreich/nützlich finden?

Zurück | Weiter

**English Translation of narrative explanation:**

*In the following section, you will be asked questions regarding a proposed "Digital Toolbox" app. The intended purpose of the app is to provide clinical professionals and students access to a library of openly accessible tools for digital learning and digital therapy. Such a tool could potentially contribute to further knowledge sharing regarding digital tools in our interdisciplinary fields and to greater quality assessment of such tools in the future.*

## App-relevante Fragen 2

*Wie würden Sie die folgenden Funktionen einer solchen App bewerten?

| | nicht sinnvoll | wenig sinnvoll | sinnvoll | sehr sinnvoll |
|---|---|---|---|---|
| Einführungs-Tutorial | ○ | ○ | ○ | ○ |
| Filterfunktion basierend auf ***Inhalt*** | ○ | ○ | ○ | ○ |
| Filterfunktion basierend auf ***Zweck/Fokus*** | ○ | ○ | ○ | ○ |
| Filterfunktion basierend auf ***Format*** | ○ | ○ | ○ | ○ |
| Filterfunktion basierend auf ***Sprache*** | ○ | ○ | ○ | ○ |
| Filterfunktion basierend auf ***Quellen (z.B. Universität, kommerziell)*** | ○ | ○ | ○ | ○ |
| Filterfunktion basierend auf ***beabsichtigtes Bildungsniveau / Zielgruppe*** | ○ | ○ | ○ | ○ |
| Möglichkeit, Tools zu bewerten (z. B. Bewertung mit Sternen, Kommentare hinterlassen) | ○ | ○ | ○ | ○ |
| Glossar mit E-Learning- und E-Health-relevanten Begriffen | ○ | ○ | ○ | ○ |
| eine App-Community (Kommunikationsfunktion) | ○ | ○ | ○ | ○ |

Fallen Ihnen noch weitere Funktionen ein?

*Wie würden Sie die folgenden ***technischen Steuerungsfunktionen*** bewerten?

| | nicht sinnvoll | wenig sinnvoll | sinnvoll | sehr sinnvoll |
|---|---|---|---|---|
| Möglichkeit, das Tool zu teilen (z. B. per E-Mail, Whatsapp, soziale Netzwerke) | ○ | ○ | ○ | ○ |
| Möglichkeit, Tools in eigenen Kategorien und Ordnern zu verwalten / zu speichern | ○ | ○ | ○ | ○ |
| Benutzername und Passwort Login | ○ | ○ | ○ | ○ |
| Benachrichtigungen zu Updates oder neuen Tools | ○ | ○ | ○ | ○ |
| Funktion zur Meldung technischer Probleme | ○ | ○ | ○ | ○ |

Fallen Ihnen noch weitere technische Steuerungsfunktionen ein?

Zurück

Weiter

## App-relevante Fragen 3

*Wie wichtig sind die folgenden Themenbereiche für Sie?

| | gar nicht wichtig | nicht wichtig | wichtig | sehr wichtig |
|---|---|---|---|---|
| Anatomie und Physiologie | ○ | ○ | ○ | ○ |
| Pathologie | ○ | ○ | ○ | ○ |
| Diagnostik | ○ | ○ | ○ | ○ |
| Therapie | ○ | ○ | ○ | ○ |
| Berufsausübung | ○ | ○ | ○ | ○ |
| Professionelles Netzwerk | ○ | ○ | ○ | ○ |

*Welche Schwierigkeiten oder Gründe sprechen Ihrer Ansicht nach gegen eine „Digital Tool Box" App?

Bitte wählen Sie die zutreffenden Antworten aus:

- ☐ Keine Bedenken
- ☐ Technische Schwierigkeiten
- ☐ Sicherung meiner persönlichen Daten, Datenschutz
- ☐ Ich bezweifle die Nützlichkeit einer solchen App
- ☐ Sonstiges: ______

Zurück | Weiter

## Gesamteindruck 2

Haben Sie weitere Vorschläge oder Kommentare zur Umfrage?

Zurück | Absenden
